# Quality- and Health-Promoting Compounds of Whole Wheat Bread with the Addition of Stale Bread, Cornmeal, and Apple Pomace

**DOI:** 10.3390/foods13111767

**Published:** 2024-06-05

**Authors:** Dorota Gumul, Joanna Oracz, Dorota Litwinek, Dorota Żyżelewicz, Tomasz Zięba, Renata Sabat, Anna Wywrocka-Gurgul, Rafał Ziobro

**Affiliations:** 1 Department of Carbohydrate Technology and Cereal Processing, Faculty of Food Technology, University of Agriculture in Krakow, 122 Balicka Street, 30-149 Krakow, Poland; dorota.litwinek@urk.edu.pl (D.L.); renata.sabat@urk.edu.pl (R.S.); anna.wywrocka-gurgul@urk.edu.pl (A.W.-G.); rafal.ziobro@urk.edu.pl (R.Z.); 2 Institute of Food Technology and Analysis, Faculty of Biotechnology and Food Sciences, Lodz University of Technology, 2/22 Stefanowskiego Street, 90-537 Lodz, Poland; joanna.oracz@p.lodz.pl (J.O.); dorota.zyzelewicz@p.lodz.pl (D.Ż.); 3 Department of Food Storage, The Faculty of Life Sciences and Technology, Wrocław University of Environmental and Life Sciences, Chełmońskiego 37, 51-630 Wrocław, Poland; tomasz.zieba@upwr.edu.pl

**Keywords:** fortified bread, secondary raw materials, fruit waste, extruded preparations, bread quality, antioxidants, vitamin B, physical properties

## Abstract

The aim of this study was to evaluate the effect of extruded preparations on the bioactive and nutritional properties, vitamin B content, volatile compound profile, and quality of whole wheat bread. Extruded preparations based on stale bread (secondary raw materials) and apple pomace (byproducts) were used as bread additives. It was found that the preparations did not enrich the bread in protein but in health-promoting compounds, especially gallic acid, protocatechuic acid, caffeic acid, p-coumaric acid, rutin, quercetin, and B vitamins. Extruded preparations had a positive effect on the quality of the bread produced, such as yield and cohesiveness, and gave it a pleasant aroma. It was shown that among all the examined bread samples with added extruded preparations of stale bread, the cornmeal and apple pomace bread samples with 15% extruded preparation (containing 55% cornmeal, 30% stale bread, and 15% apple pomace) had sufficient nutritional value, the highest amounts of gallic acid, protocatechuic acid, p-coumaric acid, caffeic acid, rutin, and quercetin; medium amounts of ellagic acid; high antioxidant activity determined in vitro using four methods (by DPPH, ABTS, power (FRAP), and Fe(II) chelating assays); adequate quality; and significant amounts of vitamins, especially B1, B2, and B3. This type of extruded preparation should utilize apple pomace, which is a byproduct, and stale bread, which is a secondary waste. Such a combination is an excellent low-cost, easy, and prospective solution for the baking industry that could be applied to obtain bread with elevated nutritional value and enhanced health potential, as proven in this publication.

## 1. Introduction

Bread is a key element in people’s daily diets. However, it is a perishable starch product that undergoes aging, making it unfit for consumption. Additionally, part of the bread in a bakery that does not meet standards can also be treated as waste, which bakeries most often use to produce breadcrumbs for the coating of other products. Estimated losses of bakery products range from 7 to 10% of their total production. Taking into account the estimated global annual production of baked goods, which in 2011 was about 125 million tons, the amount of waste worldwide could reach as much as 12.5 million tons per year [1,2,3]. In the U.K. alone, bread waste is the second food waste product after potatoes [2]. The results from the Polish bakery and confectionery industry presented by Goryńska-Goldmann et al. [4] indicate that total losses amounted to around 2.5% in terms of product weight, being relatively low in comparison with other countries. Bread leftovers account for a significant portion of food waste from retail sales, thus becoming a serious environmental challenge and an economic loss for the food sector. 

Utilization of bread waste is possible by producing biotechnology products like ethanol, enzymes, lactic acid, succinic acid, and even aromatic compounds using fermentation by the yeast *Geotrichum Candidum* after three extraction methods (headspace sampling, cold finger capture, and solid phase capture) [3,5,6,7,8].

According to Kawa-Rygielska et al. [9] bread waste is an attractive source for bioconversion into value-added products. In the work of the above-mentioned authors, the edible filamentous fungi *Neurospora intermedia* and *Aspergillus oryzae* were used for the production of bioethanol and high-protein biomass by culturing on enzymatically liquefied bread waste medium at a concentration of 150 g/L dry basis. The aforementioned authors demonstrated that the valorization of bread remnants by fungi is a promising option for biofuel and food production within a closed-loop bioeconomy. The above-mentioned possibilities for bread waste management are in the sphere of biotechnology. 

A few publications in the last few years have considered the use of bread waste in food technology. A study by Garcia-Hernandez et al. [10] explored the possibility of using bread waste in the form of flour as a replacement for wheat flour at 20, 40, 80, and 100 percent to make white bread. It was shown that the 20% share of stale bread in the re-baking was the most beneficial, as it had comparable color, volume, and texture as well as starch digestibility as the control bread, while the higher percentage of replacing wheat flour with flour from stale bread precipitation caused a deterioration in the quality of bread. The study by Meral and Karaoğlu [11] also used the process of recycling old bread for baking different types of wheat bread and investigated their physical, textural, and sensory characteristics. It was shown that using only 15% stale ground bread, which replaced flour in the recipe, provided high-quality bread. The authors stated that this was a significant percentage, taking into account the amount of waste bread recycled each year. Immonen et al. [12] were of the opposite opinion, as they showed that the addition of old bread to new wheat dough reduces the volume of baked bread and increases crumb hardness and the staling rate, which are important parameters of wheat bread quality. These authors gave two reasons for the deterioration of bread quality. First, gelatinized starch in old bread can hinder the formation of an optimal gluten network through physicochemical interactions and bind large amounts of water from the dough, thus preventing the proper hydration and elasticity of the gluten. The second reason was that the denatured gluten proteins in the old bread, added to the baking of the new one, could not participate in the formation of the new gluten network, thus reducing the proportion of active and native protein in the dough. Studies by Savkina et al. [13] showed that the addition of 25% recycled old bread into sourdough affected bread quality. The quality of this type of bread was compared to the control, and the crispness was 1.5 times lower than the control bread, indicating a slowdown in the staling process of bread produced with the addition of 25% recycled old bread to sourdough. Bread with 25% recycled ground bread into the sourdough had sensory characteristics (such as shape, the surface and color of the crust, taste, smell, chewiness, and porosity) comparable to the control. Savkina et al. [13] noted that based on their research results, they developed a new technology for processing old bread that allows for an increase in the amount of bread recycled compared with existing methods. On the other hand, Weegels [14] prepared sourdough from one-day-old bread and found that it can be used as the raw material for baking bread. He observed that white bread made with sourdough from one-day-old white bread was softer in texture and exhibited a good smell and taste. The consumer panel gave better scores to the crumb color of the standard bread (without sourdough), but because of its taste and softness, the white bread with sourdough was preferred over the standard. Sisman et al. [15] explored another application of stale bread by extracting proteins from stale bread using an isoelectric method to increase the volume of wheat bread containing chickpea flour with this type of protein. An isoelectric method was used to isolate 10% protein from the stale bread and then produce a powder. This powdered protein extract showed similar reference spectra to gluten. It was added in two levels, i.e., 6.5% and 13%, resulting in a 10% and 20% increase in the volume of wheat bread containing chickpea flour (wheat 60% and chickpea flour 40%, respectively). Another possibility for stale bread application was suggested in a study by Samray et al. [3] using the extrusion process. Samray et al. [3] attempted to use old bread in the production of cereal snacks that are very popular among many age groups. In contrast, in the present study, we attempted to produce extrudates from dry wheat bread, fruit (apple) pomace, and cornmeal, in order to reuse them in bread production after grinding. This is an innovative bread-making alternative, where part of the flour is replaced with extrudates made with dry wheat bread and apple pomace instead of producing bread in which part of the flour is replaced with stale bread, as performed by the above-mentioned researchers [10,11,13]. 

Therefore, this study aims to investigate the effect of three different levels (5, 10, and 15%) of two formulations with different percentages of secondary carbohydrate raw materials on the chemical composition of the resulting bread, especially the content of bioactive compounds including polyphenols and B vitamins, and to show their correspondence to the antioxidant activity of whole grain bread. In addition, texture, color, and volatile profile analyses were carried out to confirm the appropriate quality of the innovative final product obtained. 

## 2. Materials and Methods

### 2.1. Materials

The material for baking wheat bread was wheat flour type 1850, which was replaced with two extruded formulations based on cornmeal, carbohydrate secondary products (ground dried wheat bread), and apple pomace in the amount of 5, 10, and 15%.

#### 2.1.1. Manufacture of Extrudates from Recycled Materials—Preparations P1 and P2

The raw materials used for extrusion were cornmeal (Sante, Warsaw, Poland), dried wheat bread from a local production facility (Handelek, Krakow, Poland), and apple pomace supplied in dried form from a local fruit processor (Hortino, Lezajsk, Poland).

Extrusion was carried out in a 20DN single-screw laboratory extruder (Brabender, Duisburg, Germany), using the following parameters: screw speed—200 rpm, nozzle diameter—4 mm, compression ratio—1:3, and temperature profile—100–120–140 °C. Before extrusion, the different premixes were preconditioned to a moisture content of 14.5%. Two extruded formulations with the best pro-health potential, based on cornmeal, carbohydrate secondary products (ground dried wheat bread), and apple pomace as a byproduct, were used with the composition of formulation 1 (P1) 55/30/15 and formulation 2 (P2) 40/40/20, respectively.

#### 2.1.2. Bread Baking

All the ingredients, quantified according to Table 1, were placed in a mixer at the same time and combined together. After mixing the recipe ingredients in a spiral mixer (type SP12, Diosna Dierks & Söhne GmbH, Osnabrück, Germany), the dough was left at 40 °C for 30 min in order to multiply the yeast and rest the dough. Dough pieces weighing 150 g were then formed manually and fermented for 40 min in the proofing chamber of an oven (MIWE Condo electric oven type CO 2.0608, MIWE GmbH, Arnstein, Germany). The bread was baked in aluminum pans at 230 °C for 30 min (for the first 5 min in the steamed chamber, after which time, the steam was drained and the bread samples were refined for 25 min). All types of bread were baked at least twice. The results are presented as the average of the repetitions and different batches of bread.

### 2.2. Chemical Reagents

Phenolic standards, including catechin, rutin, caffeic acid, chlorogenic acid, p-coumaric acid, 2,5-dihydroxybenzoic acid, ellagic acid, ferulic acid, gallic acid, p-hydroxybenzoic acid, protocatechuic acid, vanillic acid, syringic acid, sinapic acid, 3,4-di-O-caffeoylquinic acid, 6-hydroxy-2,5,7,8-tetramethylchroman-2-carboxylic acid (Trolox), (2,2′-azino-bis(3-ethylbenzothiazoline-6-sulfonic acid) (ABTS), 2,2-diphenyl-1-picrylhydrazyl (DPPH), 2,4,6-tri(2-pyridyl)-s-triazine (TPTZ), sodium acetate, disodium ethylenediaminetetraacetate dihydrate, ferric chloride hexahydrate, ferrozine, ammonium acetate HPLC-grade acetonitrile (≥99.9%), and formic acid for LC-MS (~98%) were from Sigma-Aldrich (St. Louis, MO, USA). Water was purified using the Milli-Q water purification system (Millipore Corp., Bedford, MA, USA). All other chemicals were of analytical quality, and the reagents were prepared according to standard analytical procedures.

### 2.3. Chemical Composition 

#### 2.3.1. Proximate Composition

Protein content (N’5.7) was determined by the Kjeldahl method (AOAC method no. 920.87) using a Kjeltec 2200 extraction device (Foss, Hillerød, Denmark), fat content by the Soxhlet method (AOAC method no. 953.38) using a Soxtec Avanti 2055 device (Foss, Hillerød, Denmark), and ash content (AOAC method: 920.183) according to AOAC [16]. The moisture content of the bread crumb was determined by subjecting the sample to drying for 60 min at 130 °C, following the AOAC method 925.10 (AOAC (2006). This analysis was conducted both on the day of baking and after 48 h of storage.

#### 2.3.2. Dietary Fiber Content

The dietary fiber (DF) content of non-starch polysaccharides, i.e., soluble (SDF) and insoluble (IDF) dietary fiber, was determined using the enzyme-gravimetric method [17]. Total dietary fiber (TDF) was calculated as the sum of the soluble and insoluble fractions. To remove starch and protein, ground samples were dispersed in water and treated with alpha-amylase, protease, and glucosidase. For TDF, the enzyme digest was treated with alcohol to precipitate soluble dietary fiber before filtration, and the TDF residue was washed with alcohol and acetone, dried, and weighed. For IDF and SDF, the enzyme digest was filtered, and the residue (IDF) was washed with warm water, dried, and weighed. For SDF, the combined filtrate and washings were precipitated with alcohol, filtered, dried, and weighed. The residue values for TDF, IDF, and SDF were corrected for protein, ash, and a blank sample.

### 2.4. Bioactive Compounds and Antioxidant Activity

#### 2.4.1. Preparation of Extracts 

Samples weighing 0.6 g were dissolved in 30 mL of 80% ethanol and shaken in the dark for 2 h (electric shaker: type WB22, Memmert, Schwabach, Germany), and then the precipitates were removed for 15 min at 1050× *g* using a centrifuge (type MPW-350, MPW MED Instruments, Warsaw, Poland). The supernatant was decanted and stored at −20 °C for further analysis.

#### 2.4.2. UHPLC-DAD Analysis of Phenolic Compounds

The UHPLC-DAD analysis was conducted utilizing a UHPLC+ Dionex UltiMate 3000 liquid chromatographic system equipped with a diode array detector featuring multiple wavelengths (Thermo Fisher Scientific Inc., Waltham, MA, USA). Phenolic compound separation occurred on an Accucore™ C18 column (2.1 × 150 mm, 2.6 μm particle size; Thermo Scientific, PA, USA) maintained at 30 °C. The mobile phase and gradient program followed the description by Oracz et al. [18] with some adjustments. Initial conditions were held for 7 min for column re-equilibration. Chromatograms were recorded at 280 nm for hydroxybenzoic acids and their derivatives and at 320 nm for hydroxycinnamic acids and their derivatives. The sample injection volume was 3 μL. In this study, gallic acid, protocatechuic acid, ellagic acid, vanillic acid, p-hydroxybenzoic acid, syringic acid, caffeic acid, ferulic acid, p-coumaric acid, chlorogenic acid, sinapic acid, 3,4-di-O-caffeoylquinic acid, and 2,5-dihydroxybenzoic acid were quantified using corresponding reference standards. Protocatechualdehyde was calculated based on the protocatechuic acid standard curve. Phenolic compound content was expressed as mg per 100 g of the sample (mg/100 g d.m.).

#### 2.4.3. UHPLC-ESI-MS Analysis of the Vitamin B Group

B vitamins were analyzed using the UHPLC-ESI-MS method according to the procedures previously described [19,20]. Briefly, 1 g of a ground sample was extracted with a mixture of acetonitrile–acetic acid–water (5:1:94, *v*/*v*/*v*) for 40 min at 70 °C in an ultrasonic bath. After extraction, the samples were cooled to room temperature and centrifuged (10 min; 4500× *g*; 20 °C). The supernatant was filtered through a syringe membrane filter (0.2 µm) into 1.5 mL autosampler vials and analyzed by UHPLC-ESI-MS/MS. The analysis of the vitamin B group including thiamine (vitamin B1), riboflavin (vitamin B2), nicotinamide (vitamin B3), and pyridoxine (vitamin B6) in the samples was carried out using a Q Exactive™ Hybrid Quadrupole-Orbitrap™ mass spectrometry system coupled to a Thermo Scientific™ Transcend™ TLX-1 high-resolution UHPLC liquid chromatograph (Thermo Scientific, Hudson, New Hampshire, USA) according to Zohora et al. [20] with some modification. An Acclaim™ Polar Advantage II HPLC column (2.1 × 150 mm, 3 µm) was used for the separation of B vitamins. The following separation parameters for the analyzed compounds were used: column temperature—40 °C, mobile phase flow—0.25 mL/min, gradient elution, mobile phase A—0.015% formic acid in water, and mobile phase B—mixture of methanol and acetonitrile (*v*/*v*, 20:80). The following gradient was used: 0–4 min, 0% B; 4–10 min, 0–92% B; 10–11 min, 92–100% B; 11–12 min, 100% B; 12–15 min, 100–0% B; and 15–18 min, 0% B. The injection volume was set to 10 μL. The following conditions for ESI-MS/MS analysis were used: capillary voltage—4000 V in positive ion scanning mode, capillary temperature—250 °C, gas drying temperature—400 °C; drying and collision gas—nitrogen, drying and collision gas flow—10 and 8 L/min, and collision energy—25 eV. Full MS and MS2 fragmentation spectra were monitored in the *m*/*z* range of 50 to 750. MS2 fragmentation spectra were obtained in Parallel Reaction Monitoring (PRM) mode using high-resolution collision dissociation (HCD). Qexactive Tune 2.1, Aria 1.3.6, and Thermo Xcalibur 2.2 software were used to control, record, and analyze the obtained results. The identification of B vitamins was based on a comparison of retention times, full mass spectra (ESI-MS), and fragmentation spectra (MS/MS) of analytes with available standards. The external standard method was used to determine the concentration of individual vitamins. All measurements were made in triplicate.

#### 2.4.4. Total Phenolic Content (TPC)

Total phenolic content (TPC) was determined by the spectrophotometric method using Folin–Ciocalteu reagent, according to Singleton et al. [21]. In a 50 mL volumetric flask, the ether extract was diluted 10 times with distilled water. Then, 5 mL of the extract was combined with 0.25 mL of Folin–Ciocalteau reagent and 0.5 mL of 7% Na_2_CO_3_. The contents were vortexed (WF2, Janke and Kunkel, Staufen, Germany) and stored for 30 min in the dark. Absorbance was measured using a Helios Gamma 100–240 spectrophotometer (Runcorn, UK) at λ = 760 nm. The results were expressed as mg catechin/100 g d.m. 

#### 2.4.5. Total Flavonoid Content 

Flavonoids were determined by the method proposed by El Hariri et al. [22]. First, 0.5 mL of ethanol extract was taken into a test tube, and then 1.8 mL of distilled water and 0.2 mL of 2-aminoethyldiphenylborate reagent were added. The contents of the tube were vortexed (Vortex type WF2, Janke & Kunkel, Staufen, Germany), and the absorbance was measured using a spectrophotometer (Helios Gamma, 100–240, Runcorn, England) at λ = 404 nm. At the same time, a blank was performed by mixing 0.5 mL of 80% ethanol, 1.8 mL of distilled water, and 0.2 mL of 2-aminoethyl diphenylborate reagent. The flavonoid content was expressed as mg rutin/100 g d.m.

#### 2.4.6. Antioxidant Activity Determined by ABTS and DPPH Assays

Antioxidant activity was determined using ABTS by the spectrophotometric method [23]. The radical scavenging activity was expressed as the equivalent antioxidant capacity of Trolox (mg Tx/g d.m. of the sample). Trolox solutions used for the calibration curve were in the concentration range of 0–2.5 mM (R^2^ = 0.996).

The free radical scavenging activity of the samples was also measured using DPPH by the spectrophotometric method of Sánchéz-Moreno et al. [24]. The results were expressed in mg Tx/g d.m. of samples. Trolox (6-hydroxy-2,5,7,8-tetramethylchroman-2-carboxylic acid) was used as a standard (10–100 mg/L; R^2^ = 0.989).

#### 2.4.7. Ferric Ion-Reducing Antioxidant Power 

The ferric ion-reducing antioxidant power was determined by the method of Oracz and Zyzelewicz [25]. Trolox was used as a standard (0.01–0.20 μM/L; R^2^ = 0.991), and the results were expressed in µM Tx/g d.m. of samples.

#### 2.4.8. Fe(II) Chelating Activity

The Fe(II) chelating ability of the samples was measured using the ferrozine method according to Oracz and Zyzelewicz [25]. Disodium ethylenediaminetetraacetate dihydrate (EDTA) was used as a standard (2.4–80 mg/L; R^2^ = 0.994), and the results were expressed in mg EDTA/g d.m. of samples.

### 2.5. Bread Quality Analysis 

After 2 h cooling, the yield and the volume of the bread were measured.

#### 2.5.1. Bread Yield 

The mass of the loaf was measured using a laboratory scale. Bread yield was computed using the following formula:

Bread yield = (mb/mf) × 100%(1)

where mb is the weight of the cold bread and mf is the weight of the flour used to prepare the dough formed for baking.

#### 2.5.2. Bread Volume

The bread volume was determined through a three-dimensional analysis employing a low-frequency, high-precision laser Volscan Profiler (Stable Microsystems, Godalming, UK). For this study’s bread size, a vertical step size of 2 mm and a rotational speed of 0.5 rps were utilized.

### 2.6. Analysis of Texture Parameters

Texture parameters were assessed utilizing a texture analyzer, TA.XT Plus (Stable Microsystems, UK), following a standard program at a compression rate of 5 mm/s. A slice of bread crumb, extracted from the central part of the loaf with a height of 15 mm, was compressed to achieve 50% height using a P/20 aluminum compression plate, in two cycles with a 5 s delay. The resulting Texture Profile Analysis (TPA) parameters, including hardness and cohesiveness of the crumb, served as indicators of textural properties. Calculations were executed using the accompanying software, Texture Exponent 5.1.2.0 (Stable Microsystems, UK). The analysis was conducted both on the day of baking and after 48 h of storage.

### 2.7. Color Measurement

Bread color was analyzed with a CM-5 spectrophotometer (Konica Minolta) using D65 illuminant, SCE mirror component, and 10° secondary observer. The crumb was analyzed after the loaf was cut in half, using a 30 mm aperture. The results were expressed as an average in the CIE L*a*b* color system. Differences in color between the control sample and bread samples with the preparation were expressed as ΔE.

### 2.8. Analysis of Volatile Compounds Using an Electronic Nose

Analysis of volatile compounds was carried out using the HERACLES II electronic nose (Alpha MOS, Toulouse, France) according to the procedure described by Kowalski et al. [26].

### 2.9. Statistical Analysis

To evaluate the importance of variations among the means, we employed a one-way analysis of variance (specifically, Duncan’s post hoc test) on the experimental data. This analysis was conducted at a confidence level of 0.05 using Statistica v. 8.0 software by Statsoft, Inc., based in Tulsa, OK, USA. Additionally, Pearson’s correlation coefficients were computed to discern relationships among the selected parameters.

## 3. Results and Discussion

### 3.1. Characteristics of P1 and P2 Preparations and the Wheat Flour Type 1850

The wheat flour (type 1850) used to bake the wheat bread samples contained only phenolic acids such as 2,5-dihydroxybenzoic acid (1.24 mg/100 g d.m.), protocatechuic aldehyde (7.84 mg/100 g d.m.), sinapic acid (0.98 mg/100 g d.m.), and ferulic acid (0.20 mg/100 g d.m.) and no flavonols or flavanols. In addition, the flour did not contain vitamins B6 or B1 and had lower amounts of B3 (0.39 mg/100 g d.m.) and B2 (1.67 mg/100 g d.m.) compared with the additives, i.e., preparations extruded from carbohydrate secondary raw materials that were used to bake wholemeal bread. Total phenolic content (TPC) was 130.57 mg catechin/100 g d.m. flavonoids (35.5 mg rutin/100 g d.m.); the antioxidant activity was ABTS 16.07 mg Tx/g d.m., DPPH 1.72 mg Tx/g d.m., FRAP 3.45 µMTx/g d.m.; and the Fe(II) chelating ability was 0.35 mg EDTA/g d.m. The chemical composition of wheat flour (type 1850) was protein 12.42 g/100 g d.m., fat 1.73 g/100 g d.m., ash 1.85 g/100 g d.m., non-soluble fiber fraction 9.0 g/100 g d.m., soluble fiber fraction 2.07 g/100 g d.m., and total fiber 11.07 g/100 g d.m.

In the first stage of this study, two extruded formulations based on cornmeal, carbohydrate secondary products (ground stale bread—ground light wheat bread dried in a dryer at about 80 °C containing less than 10% moisture, 3% fat, 75% carbohydrates, and 11.7% protein), and byproducts (apple pomace) were also analyzed as follows: formulation 1 (P1) 55/30/15 and formulation 2 (P2) 40/40/20. Formulation P1 contained more protein, fat, ash, and total dietary fiber (including its soluble insoluble fraction) than formulation P2 by 3%, 15%, 17%, and 17%, respectively. In a similar study by Samray et al. [3] that compared the physical and functional properties of bread crumb extrudates (BCEs) to wheat flour extrudates (WFEs), it was shown that stale bread can be used to produce extrudates (BCEs) that have a significantly higher amount of fiber relative to WFEs.

It was found that among the analyzed preparations, P1 had the highest content of total polyphenols and flavonoids and antioxidant activity, as it contained 261.31 mg catechin/100 g d.m. polyphenols, 49.02 mg rutin/100 g d.m. flavonoids, and had the scavenging activity of ABTS+ radical oscillating at 15.15 mg Tx/g d.m. In contrast, P2 contained 213.72 mg catechin/100 g d.m. polyphenols, 39.31 mg rutin/100 g d.m. flavonoids, and its antioxidant activity was 14.51 mg Trolox/g d.m. (Table 2). The antioxidant activity estimated by DPPH was at the level of 1.89 mg Tx/g d.m. and 1.78 mg Tx/g d.m. for P1 and P2, respectively (Table 2). The FRAP results showed that the P1 formulation (8.91 µMTx/g d.m.) had a higher ferric-reducing antioxidant power than P2 (6.57 µMTx/g d.m.). Preparations P1 and P2, on the other hand, showed a similar ability to chelate ferrous ions (1.21 and 1.23 mg EDTA/g d.m., respectively, as shown in Table 2).

Analyzing the profile of phenolic compounds by UHPLC, it was observed that P1 contained 2 times more hydroxybenzoic acids than P2, such as gallic acid and protocatechuic acid, and the identical content of syringic acid and ellagic acid. It also contained vanillic acid, which was absent from P2. As for hydroxycinnamic acids, P1 contained 16% more chlorogenic acid, a comparable amount of p-coumaric acid, but 30% less ferulic acid than P2. P1 had half the amount of rutin, twice the amount of quercetin, and 30% less epicatechin relative to P2. Considering the total amount of phenolic compounds determined by UHPLC, it was found that P1 had polyphenols at a level of about 57 mg/100 g of preparation, while P2 had about 38 mg/100 g of preparation (Table 3).

The total content of B vitamins, calculated as the sum of the content of vitamins B1, B2, B3, and B6, in formulation 1 (P1) was higher than in P2 (Table 3). In addition, there was variation in the levels of individual vitamins between the formulations. In particular, the amount of vitamin B2 was 30% higher in P1 than in P2, while the content of vitamins B1 and B3 was higher in P2 than in P1 by 26% and 60%, respectively (Table 3). 

In summary, the above-mentioned preparations can be considered functional additives to whole wheat bread because of the high content of quercetin 3-O-galactoside and quercetin 3-O-rutinoside, quercetin, epicatechin, and phenolic acids (gallic, protocatechuic, ellagic, and chlorogenic acid), which are absent in the whole wheat flour. Thus, these results suggest that the respective extrudates can enrich whole wheat bread with new bioactive compounds from the group of polyphenols as well as vitamins, mainly B1 and B6, which in turn will generate a higher antioxidant potential of such products.

### 3.2. Effect of the Extruded Preparations on the Amount of Nutritional Compounds and Bioactive Compounds in Whole Wheat Bread

The second stage of this study investigated the effect of the extruded preparations based on secondary raw materials on the amount of nutritional and bioactive compounds from the polyphenol group and B vitamins as well as the antioxidant potential of whole wheat bread.

Considering the bread samples with 5 to 15% P1 and P2 extruded preparations, it was found that the amount of protein was the same as for the standard bread. The fat content of the bread samples with the addition of the P1 and P2 preparations decreased from 11 to 21% compared with the standard. Similarly, the amount of ash in the bread with the extruded P1 and P2 preparations also decreased from 7 to 11% compared with the control (Table 4).

The smallest amount of insoluble fiber fraction was recorded in the bread with 5% of the P2 formulation, and the highest amount was in the bread with 10% of the P1 formulation. In contrast, the other fortified bread samples and the control contained identical amounts of this insoluble fiber fraction. It was found that the soluble fiber fraction decreased in the bread samples after the addition of the extruded formulations compared with the control bread. The content of total fiber was highest in the bread samples with 10% of the P1 formulation and lowest with 5% of the P2 formulation, which was a consequence of having the lowest content of insoluble fiber fraction (Table 4).

It should be acknowledged that the chemical composition of the bread both with and without the preparations was determined by the use of whole wheat flour (type 1850) for baking, as it contained significantly higher amounts of all nutrients, including dietary fiber, in relation to the preparations acting as fortifying additives. It was observed that the preparations used did not enrich the bread with these nutrients. A different trend was found when analyzing the bioactive compounds from the polyphenol group and vitamin B in the bread fortified with the extruded preparations. It was found that the bread samples fortified with the formulations had a higher content of total polyphenols, ranging from 10 to 48%, compared with the control, with the exception of the bread with 5% P1. It was also observed that although formulation P1 had a higher amount of polyphenols than formulation P2 (Table 2), the bream samples fortified with it showed a lower amount of total polyphenols (Table 4). We can explain this tendency by the Maillard reaction products (MPRs) that were present in greater quantity in formulation P2 than in formulation P1. Therefore, the amount of MPRs increases sharply when baking the bread with formulation P2. And, as it is well known, the Folin–Ciocalteau reagent reacts not only with polyphenols but also with vitamin C, alkaloids, sugars, and amino acids [27,28,29,30]. The latter two form the products of the Maillard reaction; hence, their high amount in the bread samples involving formulation P2 in comparison with P1 (Table 4). The low polyphenol content of bread with 5% P1 can be explained in a similar way.

Considering the content of total flavonoids, their amount increased in the bread samples with the P1 formulation by 2–3 times, and in the case of the bread samples with the P2 formulation, from 43 to 84% compared with the control. It should be noted that the 5 and 10% share of the P1 and P2 formulations caused an increase in flavonoids in the bread samples at an identical level compared with the control, and only a 15% share of the above-mentioned formulations contributed to a significant increase in these bioactive components in fortified bread samples (Table 4).

The quantitative and qualitative profile of phenolic compounds by UHPLC chromatography was also analyzed. It was noted that the total content of phenolic compounds in the bread fortified with the preparations increased by an average of 22% compared with the control (Table 5). In the control wheat bread, there was a significant content of phenolic acid derivatives that are present in the wheat flour used for baking, i.e., ferulic acid, sinapic acid, 2,5-dihydroxybenzoic acid, and protocatechuic aldehyde. This is in agreement with results from other authors, who noted that wheat flour is a source of the aforementioned acids [31,32,33].

Replacement of this flour with byproduct extrudates, which are characterized by the absence of sinapic acid, 2,5-dihydroxybenzoic acid, and protocatechuic aldehyde, resulted in a decrease in their content in the wheat bread with these additives. On the other hand, an increase in the other phenolic acids, especially hydroxybenzoic acids (gallic, caffeic, p-coumaric, protocatechuic, and ellagic acid) along with quercetin and rutin, in the wheat bread samples with the addition of the byproduct extrudates is the result of the use of such additives in the baking of the final products (Table 5). In the case of vanillic acid and chlorogenic acid, their decrease was observed in the bread samples with the extruded formulations because of the thermal decarboxylation of these compounds to, among others, 4-vinyl guaiacol [34] during baking. Another reason could be the decomposition of the ester bonds, as observed for chlorogenic acid, which could be decomposed into quinic and caffeic acids. Thus, the amount of caffeic acid increased in the bread samples with the P1 and P2 preparations. Although they initially did not contain the above-mentioned acid, they were rich in chlorogenic acid (Table 3 and Table 5). Ferulic acid, although present in the extruded preparations with byproducts, is mainly derived from the whole-grain flour used in bread baking, so its amount decreases in parallel with the level of addition in the bread samples with the preparations in relation to the control (Table 5).

The content of syringic acid and ellagic acid in the bread samples with the extruded byproducts was higher than the expected increase resulting from the percentage of these byproducts (Table 5). This is most likely related to the different stages of bread production since, according to Katina et al. [35], the amount of phenolic acids increases during the yeast fermentation process, as well as during dough mixing [36]. In addition, this may in part be due to the thermal breakdown of quercetin derivatives, especially quercetin 3-O-rutinoside, which generates phenolic acids [32,37,38]. Given that the amount of quercetin derivatives in the extruded byproducts is high (Table 3), their thermal degradation may in part contribute to the increase in the amount of phenolic acids in byproduct bread, as evidenced by the fact that there was a large increase in two phenolic acids (syringic acid and ellagic), inadequate to the increasing proportion of ground byproduct in these bread samples (Table 5). In the case of gallic acid, it was observed that its amount increased in the whole wheat bread samples parallel to the level of extrudate addition. 

On the other hand, the content of flavan-3-ols, which include epicatechin, in the bread samples with the extruded byproducts was beyond determination (Table 5). This is most likely related to the fact that there was a significant reduction in these compounds, which may be the result of a combination of several processes, i.e., oxidation, isomerization, epimerization, and their degradation both during baking [39] and other stages of bread production [32]. In addition, losses of these phenolic compounds can be caused by the formation of complexes with polysaccharides [39,40].

Thus, it can be suggested that the stability of phenolic compounds in food products may be affected by a variety of factors, ranging from several mechanisms of polyphenol degradation during thermal processes to the ingredients in product formulations.

In conclusion, it should be said that the baking process affects the loss of some phenolic compounds [41,42], in this case, especially phenolic acids (p-coumaric, chlorogenic, vanillic, protocatechuic) and quercetin derivatives (Table 5). According to the above-mentioned authors, losses in these compounds can reach up to 60%. These losses are influenced by a great number of factors such as thermal, enzymatic, and oxidative degradation, as well as the isomerization/epimerization and decarboxylation processes of phenolic acids mentioned earlier [41,43]. Although thermal processes such as baking contribute to the loss of polyphenols, the introduced ground extruded byproduct preparations, which are a source of bioactive compounds, guaranteed a significant content of phenolic compounds in these final products, that is, in the bread samples with their addition (Table 5). It was found that the amount of gallic acid increased up to 10 times in the bread samples with the addition of ground extruded byproduct preparations compared with the standard. There were also new compounds such as phenolic acids (caffeic, p-coumaric, protocatechuic acid, ellagic acid), quercetin derivatives (quercetin 3-O-rutinoside), and quercetin in the bread samples with the addition of milled preparations compared with the standard (Table 5).

In an analogous study on gluten-free bread, Gumul et al. [44] showed that the health-promoting potential depends on the extrudates that are introduced. Analyzing gluten-free bread with extrudates obtained at two temperatures of 80 and 120 °C based on rice flour with 10 and 20% sour cherry pomace, they found that the antioxidant activity of these bread types increased by up to six times compared with the control. The amount of total polyphenols ranged from 3.10 to 308.7 mg of catechin per kilogram of bread, while the amount of flavonoids varied from 6.40 to 97.3 mg of rutin per kilogram in bread with the above preparations. In the case of phenolic acids, their amounts were 2.14 and 2.37 mg of ferulic acid per kilogram only in the two bread types with the addition of rice flour extrudates with 20% sour cherry pomace, while in the other variants, their level was below the detection limit. According to the authors, their loss resulted from thermal, enzymatic, and oxidative degradation, and, in the case of phenolic acids, decarboxylation, leading to the formation of 4-vinylguaiacol.

In general, the antioxidant activity of the whole wheat bread samples with extrudates was greater than that of the control (Table 4). The increase in ABTS-estimated antioxidant activity of the bread samples with the P1 and P2 extrudates ranged from 8 to 47% compared with the control. In the case of DPPH, a similar change in the bread samples with the formulations ranged from 5.9 to 29% compared with the control. The bread samples with the formulations also showed significant reducing activity as indicated by their much higher FRAP values (from 5.3 to 27.3%) compared with the control. On the other hand, the chelating capacity decreased with the share of 5% of the P1 and P2 preparations in the bread samples and increased with a higher share of the above preparations compared with the control. There was a strong correlation between TPC and DPPH of 0.844 and between TPC and ABTS of 0.818, as well as moderate correlations between the amount of flavonoids and DPPH of 0.539 and flavonoid content and ABTS of 0.628. The results for antioxidant properties also showed that the TPC of the bread samples tested was strongly correlated with ferric ion-reducing capacity and Fe(II) chelating activity (R2 = 0.966 and R2 = 0.737, respectively). In contrast, no correlation was found between flavonoid content and the reducing power and chelating ability of the whole wheat bread samples.

It should be unequivocally stated that among the analyzed whole grain bread samples with the P1 and P2 preparations, those with 15% of these preparations, especially P1 preparation, had the highest antioxidant activity. The high antioxidant activity of the bread with 15% of P1 can be explained by the highest content of caffeic acid and sinapic quercetin and a significant amount of ferulic acid, compounds that show the highest efficiency in scavenging DPPH and ABTS free radicals according to the authors of [45,46,47]. In addition, the contribution to the free radical scavenging activity and ferric ion reducing capacity of this sample can also be attributed to the highest content of gallic acid, protocatechuic acid, p-coumaric acid, and products of the Maillard reaction, which can be formed during technological processes, especially high-temperature processes such as extrusion and bread baking. However, despite the higher content of phenolic compounds, particularly gallic acid, only a slight increase in the iron ion chelating capacity of the extruded breads can be explained by the fact that gallic acid has a lower iron ion binding capacity than some other dihydroxy compounds [48]. In addition, some authors suggest that the presence of a methoxy group in the structure of some phenolic acids, including vanillic, syringic, and ferulic acids, limits their chelating capacity [48,49]. The chelating activity of metal ions by phenolic compounds is mainly related to the presence of ortho-dihydroxy groups in their structure. Phenolic compounds can donate a proton and act as hard Lewis bases, forming complexes with hard Lewis acids such as Fe(III) iron ions. In addition, polyphenols with ortho-dihydroxyl groups such as the catechol group can reduce Fe(III) ions to Fe(II) via the one-electron transfer pathway and are oxidized to semiquinones [49].

The fortified bread samples had a significantly higher total content of B vitamins in comparison with the standard bread. The amount of vitamin B1 increased by up to two times, B2 by up to 60%, and B6 by up to 42% in all the bread samples with the extruded preparations, especially with P1, compared with the control (Table 6). The content of vitamin B3 decreased with the share of 5% of P1 and P2 preparations in the bread samples and increased with a higher share of the above-mentioned preparations relative to the control.

### 3.3. The Effect of Extruded Preparations on the Texture and Physical Properties of Whole Wheat Bread

Whole wheat bread is generally characterized by inferior physical properties of bread. This is a result of the negative effect of dietary fiber on the gluten lattice formed in wheat dough [50] and also a reduction in the ability of starch to swell and stick together in the dough during baking [51], which manifests itself as a poorer crumb structure and porosity (harder and less elastic crumb), a small loaf volume, and a darker crumb color compared with wheat bread made from light (low-coal) flour. The inferior quality characteristics make whole wheat bread unpopular with consumers despite its much higher nutritional value [52,53]. Therefore, research is constantly being conducted on the possibilities of improving the quality of whole-grain bread [52,54].

**Figure 1:**
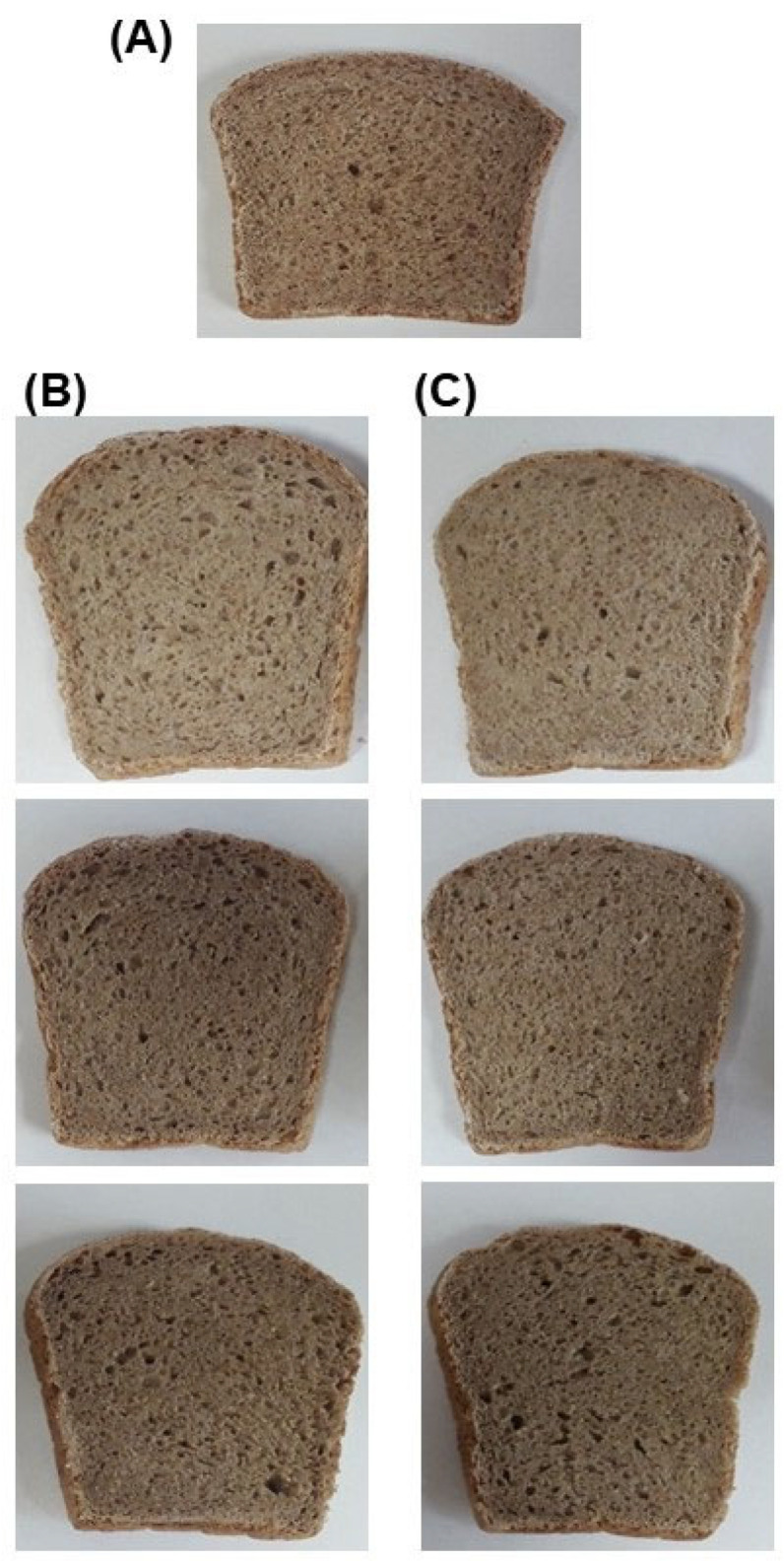
Photographs of the bread samples. (**A**) Control; (**B**) increasing P1 addition; and (**C**) increasing P2 addition.

The use of flour obtained from dried bread in baking has been studied earlier [10,11,55], but those studies focused on obtaining light-colored bread. It was found that with the increase in the share of dried bread, in general, worse quality characteristics (lower volume, worse texture) and, above all, a deterioration in the color of the product were obtained. Since whole-grain bread is characterized by a dark crumb, the addition of secondary raw materials did not have such a significant effect on the color of bread (Figure 1A–C).

As a result of this study, it was observed that the 5% share of the P1 preparation did not significantly affect the volume of the bread samples (Table 7), which were characterized by very similar parameters as the control bread. However, a slight but statistically significantly lower volume was observed in the bread samples with a 5% share of the P2 preparation and a 10% share of both the P1 and P2 preparations, compared with the control bread (Table 7). Comparing the cross-sections obtained (Figure 1B,C) for the bread samples with 5% and 10% of the preparation, there were no noticeable differences except for the color, which was significantly darker in the case of the bread with 10%, while the bread with 15% showed a less porous crumb and a significantly darker color. However, regardless of the amount of the preparation added and its type, the color was characteristic of the control whole-grain bread (Figure 1). All the bread samples with preparations were distinguished by a significantly higher bread yield, which indicates less water loss during the baking process because, regardless of the recipe used, the amount of water added was the same. However, this did not clearly affect the moisture content of the crumb, which, after 48 h of storage, stabilized at a similar level of about 44% in all the bread samples tested.

The application of the P1 and P2 preparations at 5% did not significantly affect the crumb hardness of the bread samples (Table 7), but as the proportion of preparation increased, an increase in crumb hardness was observed, which is characteristic when using various bread additives including extrudate preparations [56,57]. In contrast, the use of bread preparations increased the consistency of the crumb on the day of baking, which was particularly observed after the use of P2, which resulted in a consistency that was significantly higher compared with the control bread (Table 7). This may be due to the higher soluble fiber content (Table 4), which has a positive effect on the texture of products [58,59]. 

Therefore, it can be concluded that the 5% share of the extruded P1 and P2 preparations does not significantly affect either the specific volume or hardness on the day of baking or after 48 h because these parameters are almost identical to the control. On the other hand, a higher proportion of additives (10 and 15% of the extruded preparations) decreases the specific volume and increases the hardness of the bread samples baked with these preparations compared with the control. This study confirmed the research by Immomena et al. [12], who precisely showed that the addition of old bread to the production of new wheat bread reduces the volume of the product and increases the hardness of the crumb. The reasons identified by the authors included gelatinized starch present in dry old bread that was added to form new bread, which hindered the formation of an optimal gluten network by physicochemical interactions and absorbed a large amount of water from the dough, thus preventing the hydration and development of the gluten. The second reason for the poor quality of bread obtained with the addition of old bread is the denatured protein in the old bread, which is not involved in the formation of the new gluten network. It can therefore be suggested that the change in the starch status of stale bread (gelatinized starch instead of granular starch) added to the baking of new bread will be essential in shaping its quality characteristics. 

The changes in cohesiveness were minor; however, a slight increase in this parameter was observed on the day of baking bread with the P2 preparation. Unfortunately, after 48 h, the supplemented bread had worse cohesiveness than the standard.

The crumb of the analyzed bread samples is characterized by a dark color because of the use of whole wheat flour, which is dark itself (Table 8). The rather intense color of the preparations is due to the presence of a significant amount of dried bread and apple pomace. At the same time, the differences in the color parameters of the preparations affected the differences in the coloration of the crumb in the case of the supplemented bread samples. The bread samples with the P2 preparation were distinguished by brighter color and red saturation, as evidenced by higher L* and lower a* values. The crumb of the B5P2 bread differed the least from the standard bread, as evidenced by the lowest value of ΔE; this parameter reports the noticeability of the difference in the colors of the product. It is accepted that when ∆E is below 1, the difference in color is imperceptible to the human eye, at values of 1–3, the color difference is noticeable to an experienced observer, while when ΔE is above 3, the difference in color is visible [60]. Taking this into account, it can be concluded that the P2 formulation added in amounts of less than 15% did not cause a clear difference in the color of the crumb of the bread samples with their share compared with the standard, while even a minimal share of the P1 formulation would be noticeable by consumers. 

The change in color is partly due to the use of the recycled bread share, as well as the share of apple pomace, which generally modifies the color of bread [10,61]. But it may also be a result of the lower porosity and volume of the bread samples, as, in general, the crumb is darker the lower its porosity [62], which was also observed in the study of this work.

### 3.4. Influence of the Extruded Preparations on the Volatile Compound Profile Whole Wheat Bread Evaluated by Electronic Nose

Electronic nose analysis was used to determine the effect of fortifying the bread samples with the extruded formulations on the composition and concentration of individual volatile compounds. The results of the analysis of the content of volatile compounds in the extruded preparations are shown in Table 9.

**Figure 2:**
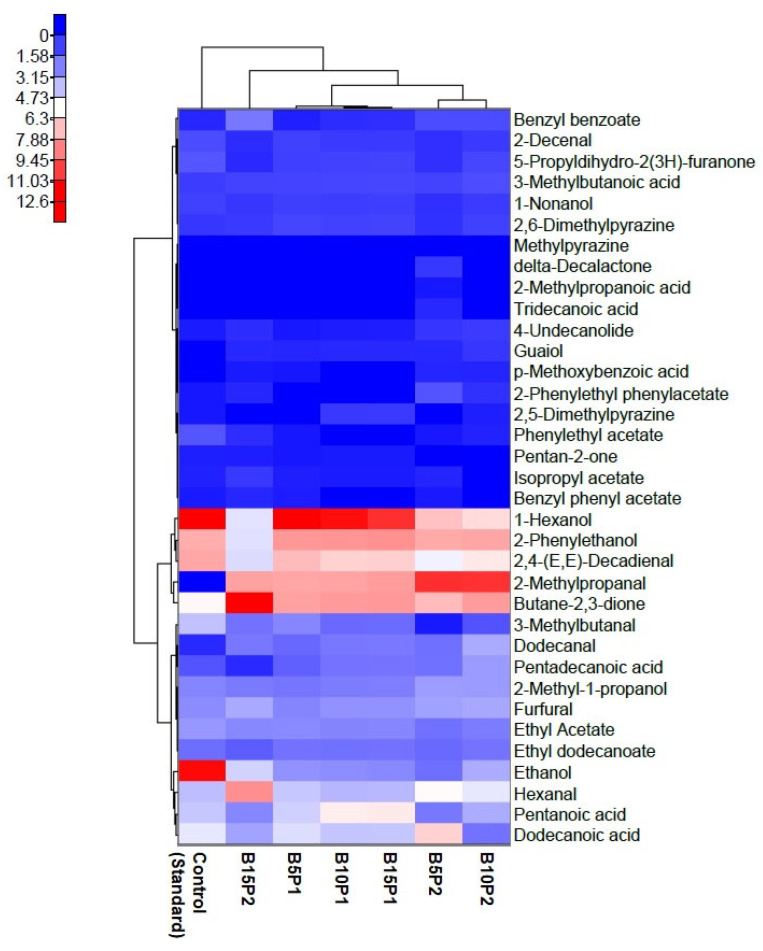
A heat map of the effect of the extruded formulations on the composition and concentration of individual volatile compounds in bread.

A total of 36 volatile compounds belonging to the groups of alcohols, aldehydes, carboxylic acids, esters, ketones, lactones, phenols, and pyrazines were determined in preparations P1 and P2. In both preparations, ethanol was the predominant volatile compound. Tridecanoic acid, 2-methyl-1-propanol, 1-hexanol, and ethyl dodecanoate were also present in significant amounts. An analysis of the volatile compound profile also showed that P1 contained twice as much dodecanal and methylpyrazine as P2, and also guaiol and 2-phenylethyl phenylacetate, which were absent in P2. On the other hand, formulation P2 contained significantly more 2,4-(E,E)-decadienal, benzyl benzoate, and 2,5-dimethylpyrazine than formulation P1. In addition, compounds absent from the P1 formulation including 2-methylpropanal, 3-methylbutanal, p-methoxybenzoic acid, isopropyl acetate, and 4-undecanolide were present in P2. The relationship between the effect of the supplementation of bread with the extruded formulations and the composition and concentration of individual volatile compounds was investigated by generating a heat map with hierarchical clusters (Figure 2).

Euclidean distance was utilized to measure the similarity among samples, while the map’s axes were used to display both the samples and volatile compounds. The varying colors in the rectangular sections indicate the concentration of various categories of aroma compounds found in the samples, with the darker red color indicating higher content and the blue color indicating a lower concentration. The results suggest that the level and profile of volatile compounds present in the bread samples are dependent on the type of additive used. Hierarchical cluster analysis (HCA) was used to categorize the bread samples into two primary clusters in the heat map. The heat map shows that the amount and type of volatile compounds found in the control (standard) bread showed some similarity to the amounts found in samples fortified with P1. It was found that the addition of P2 resulted in very large changes in the profile of volatile compounds in the bread samples as compared with the control. In the control bread, the predominant volatile compounds were ethanol and 1-hexanol. Significant amounts of 2,4-(E,E)-decadienal were also present, followed by 2-phenylethanol, butane-2,3-dione, and dodecanoic acid. The bread samples fortified with P1 were dominated by 1-hexanol, followed by 2-phenylethanol, 2-methylpropanal, and butane-2,3-dione. More 2-phenylethanol, pentanoic acid, and benzyl benzoate were found in the bread samples with a higher proportion of P1. The presence of the first two compounds can impart a pleasant aroma to bread, as phenylethanol imparts a floral and honey aroma, while benzyl benzoate imparts a sweet balsamic aroma. Pentanoic acid has a cheesy odor and sour taste and can give products an unpleasant flavor [63]. 

The profile of volatile compounds in the bread samples enriched with P2 depended on the concentration of the formulation. In the bread with the highest P2 content, butane-2,3-dione was the dominant compound. 2-Methylpropanal and hexanal were also present at relatively high levels. On the other hand, in the bread samples with lower levels of this formulation, 2-methylpropanal was the dominant compound, followed by 2-phenylethanol, butane-2,3-dione, 1-hexanol, and 2,4-(E,E)-decadienal.

## 4. Conclusions

Extruded preparations produced with cornmeal, stale bread, and apple pomace contain a high amount of quercetin 3-O-galactoside, quercetin 3-O-rutinoside, quercetin, epicatechin, and phenolic acids, such as gallic, protocatechuic, ellagic, and chlorogenic acids, which are not present in whole wheat flour. Moreover, they have significant amounts of protein and dietary fiber and can therefore serve to enrich whole wheat bread with the aforementioned health-promoting components. The extruded preparations did not enrich the bread samples with nutritional compounds such as protein, fat, ash, or fiber, with the only exception being the bread sample with 10% extruded P1. However, an increase in B vitamins (included in nutrients) was observed, especially in the contents of vitamins B1 (twice), B2 (by 60%), and B3 (by 42%), compared with the standard. The bread samples with a share of extruded preparations were characterized by a significantly higher content of the following phenolic acids: gallic acid (more than tenfold), caffeic, p-coumaric, protocatechuic, and ellagic acid, as well as rutin and quercetin compared with the control. The highest antioxidant activity was determined in the bread samples with 15% of the extruded preparations, especially P1, which is due to the highest amount of sinapic and caffeic acids and quercetin and significant amounts of ferulic acid. The applied preparations did not deteriorate the physical characteristics of the bread samples containing 5% of P1 and P2, but larger quantities were detrimental. All the preparations improved the quality parameters, increasing their yield and cohesiveness, especially 24 h after baking. The profile of flavor compounds in the bread samples obtained with the addition of the extruded preparations depended on the type of additive, which is due to the different compositions of these compounds in the preparations. 

In conclusion, it can be said that of all the analyzed bread samples, those with 15% preparation P1 were characterized by the appropriate nutritional value of the highest amount of gallic, protocatechuic, caffeic, and p-coumaric acids, rutin, and quercetin, a medium amount of ellagic acid, and the highest antioxidant activity (determined by ABTS, DPPH, FRAP, and ferrous ion chelating activity assays). The aforementioned bread samples contained a high level of vitamin B, especially B2 and B3, and exhibited a good quality and a very pleasant aroma. We suggest that this kind of formulation with extruded preparations of secondary raw materials can be used to make high-quality bread and, at the same time, reduce the amount of bakery waste, which fits perfectly into sustainability trends. The use of extruded preparations involving stale bread, apple pomace, and cornmeal in bread formulations, in which they replace part of the flour, can become an alternative to the earlier-developed bread recipes in which flour was partially replaced with ground stale bread. This type of extruded formulation can use apple pomace, which is a byproduct, and stale bread, which is a secondary waste. Such a combination is an excellent, low-cost, easy, and promising solution for the baking industry, which can be used to obtain baked goods with increased nutritional value and enhanced health potential, as proven in this publication.

## Figures and Tables

**Table 1:** Bread formulations (the amount and type of raw materials used to make the dough).

	Control * (Standard)	B5P1	B10P1	B15P1	B5P2	B10P2	B15P2
Wheat flour [g]	1000	950	900	850	950	900	850
Preparation P1 [g]	0	50	100	150	0	0	0
Preparation P2 [g]	0	0	0	0	50	100	150
Water [mL]	688	688	688	688	688	688	688
Salt [g]	20.00	19.59	19.19	18.78	19.44	18.91	18.34
Dry yeast [g]	15	15	15	15	15	15	15

* Control—control bread, B5P1—whole wheat bread with a share of 5% P1; B10P1—whole wheat bread with a share of 10% P1; B15P1—whole wheat bread with a share of 15% P1; B5P2—whole wheat bread with a share of 5% P2; B10P2—whole wheat bread with a share of 10% P2; B15P2—whole wheat bread with a share of 15% P2.

**Table 2:** Chemical composition and phenolic compounds and antioxidant activities of the preparations.

Sample	Chemical Composition (g/100 g d.m.)					
	Protein	Fat	Ash	IDF	SDF	TDF
P1	9.53 ± 0.10 b	1.17 ± 0.01 b	0.82 ± 0.03 b	3.60 ± 0.15 b	1.38 ± 0.2 b	4.98 ± 0.07 b
P2	9.28 ± 0.07 a	1.02 ± 0.02 a	0.70 ± 0.01 a	3.15 ± 0.17 a	1.10 ± 0.07 a	4.25 ± 0.04 a
	**Phenolic compounds and antioxidant activities**					
	**TPC** **(mg catechin/100 g d.m.)**	**Flavonoids (mg rutin/100 g d.m.**	**ABTS** **(mgTx/g d.m.)**	**DPPH (mgTx/g d.m.)**	**FRAP** **(µMTx/g d.m.)**	**Chel. Fe(II)** **(mg EDTA/g d.m.)**
P1	261.31 ± 3.6 b	49.02 ± 0.01 b	15.15 ± 0.07 b	1.89 ± 0.02 b	8.91 ± 0.03 b	1.21 ± 0.02 a
P2	213.72 ± 11.6 a	39.31 ± 0.27 a	14.51 ± 0.02 a	1.78 ± 0.01 a	6.57 ± 0.02 a	1.23 ± 0.01 a

Different letters in a column represent a statistically significant difference in average values (*p* ≤ 0.05). P1—extruded preparation of cornmeal, stale bread, and apple pomace at 55/30/15, respectively; P2—extruded preparation of cornmeal, stale bread, and apple pomace at 40/40/20, respectively.

**Table 3:** Quantity and quality profiles of polyphenols and B vitamins in the preparations.

Sample	Content (mg/100 g d.m.)						
	Hydroxybenzoic Acid						
	GA	VA	PA	SYA	DA	PAL	EA
P1	31.43 ± 0.13 b *	0.26 ± 0.01 a	12.26 ± 0.05 b	0.49 ± 0.01 a	nd	nd	2.47 ± 0.06 a
P2	17.32 ± 0.1 a	nd	6.12 ± 0.03 a	0.47 ± 0.03 a	nd	0.14 ± 0.01 a	2.39 ± 0.02 a
	**Hydroxycinnaminic acid**						
	**CA**	**FA**		**PCA**		**CLA**	**SIA**
P1	nd	0.22 ± 0.02 a		0.19 ± 0.01 a		3.01 ± 0.08 b	nd
P2	nd	0.29 ± 0.03 b		0.16 ± 0.03 a		2.58 ± 0.07 a	nd
	**Flavonols and flavanols**						
	**Q3Gal**	**Q3Glu**		**Q3R**		**Q**	**E**
P1	1.02 ± 0.01 a	nd		1.64 ± 0.02 a		1.74 ± 0.02 b	2.56 ± 0.07 a
P2	1.29 ± 0.02 b	nd		3.24 ± 0.04 b		0.91 ± 0.01 a	3.23 ± 0.03 b
	**B vitamins**						
	**B1**	**B2**		**B3**		**B6**	**Total**
P1	0.15 ± 0.01 a *	1.73 ± 0.01 b		0.50 ± 0.01 a		0.12 ± 0.01 a	2.50 ± 0.04 b
P2	0.19 ± 0.01 b	1.34 ± 0.01 a		0.80 ± 0.01 b		0.10 ± 0.01 a	2.43 ± 0.04 a

GA—gallic acid, VA—vanillic acid, PA—protocatechuic acid, SYA—syringic acid, DA—2,5-dihydroxybenzoic acid, PAL—protocatechuic aldehyde, EA—ellagic acid, CA—caffeic acid, FA—ferulic acid, PCA—p-coumaric acid, CLA—chlorogenic acid, SIA—sinapic acid, Q3Gal—quercetin 3-O-galactoside, Q3Glu—quercetin 3-O-glucoside, Q3R—quercetin 3-O-rutinoside (rutin), Q—quercetin, E—epicatechin; * Different letters in a column represent a statistically significant difference in average values (*p* ≤ 0.05); P1—extruded preparation of cornmeal, stale bread, and apple pomace at 55/30/15, respectively; P2—extruded preparation of cornmeal, stale bread, and apple pomace at 40/40/20, respectively. nd—Not detected.

**Table 4:** Chemical composition, phenolic compounds, and antioxidant activities of bread with a share of the preparations.

Sample	Chemical Composition (g/100 g d.m.)					
	Protein	Fat	Ash	IDF	SDF	TDF
Control (standard)	13.46 ±0.01 a *	1.89 ± 0.1 b	3.57 ± 0.01 b	9.72 ± 0.2 b	2.06 ±0.01 b	11.78 ± 0.01 d
B5P1	13.51 ± 0.10 a	1.63 ± 0.07 a	3.32 ± 0.02 a	9.65 ± 0.15 b	1.69 ±0.17 a	11.34 ± 0.12 b
B10P1	13.36 ± 0.12 a	1.59 ± 0.01 a	3.28 ± 0.03 a	10.60 ± 0.01 c	1.43 ±0.1 a	12.03 ± 0.3 e
B15P1	13.15 ± 0.01 a	1.49 ± 0.12 a	3.28 ± 0.01 a	9.76 ± 0.01 b	1.53 ± 0.16 a	11.28 ± 0.01 bc
B5P2	13.44 ± 0.02 a	1.68 ± 0.01 a	3.17 ± 0.03 a	9.05 ± 0.17 a	1.58 ± 0.02 a	10.62 ± 0.1 a
B10P2	13.22 ± 0.1 a	1.62 ± 0.13 a	3.22 ± 0.05 a	9.50± 0.2 b	1.62 ± 0.01 a	11.11 ± 0.12 b
B15P2	13.03 ± 0.2 a	1.59 ± 0.12 a	3.16 ± 0.07 a	9.68 ± 0.19 b	1.91 ± 0.01 a	11.60 ± 0.3 d
	**Phenolic compounds and antioxidant activities**					
	**TPC** **(mg catechin/100 g d.m.)**	**Flavonoids** **(mg rutin/100 g d.m.)**	**ABTS** **(mgTx/g d.m.)**	**DPPH (mgTx/g d.m.)**	**FRAP (µMTx/g d.m.)**	**Chel. Fe(II) (mg EDTA/g d.m.)**
Control (standard)	79.69 ± 1.55 b	8.83 ± 0.05 a	11.83 ± 0.14 a	1.52 ± 0.02 b	2.69 ± 0.01 b	1.22 ± 0.03 c
B5P1	62.18 ± 1.56 a	18.60 ±1.8 c	12.87 ± 0.11 b	1.47 ± 0.01 a	2.54 ± 0.03 a	1.14 ± 0.02 b
B10P1	88.44 ± 1.50 c	19.66 ± 2.1 c	12.75 ± 0.08 b	1.62 ± 0.05 c	2.98 ± 0.02 d	1.24 ± 0.01 c
B15P1	111.97 ± 2.32 e	25.31 ± 1.84 d	17.15 ± 0.71 f	1.97 ± 0.04 f	3.42 ± 0.02 f	1.31 ± 0.02 d
B5P2	87.90 ± 2.28 c	12.68 ± 1.79 b	13.62 ± 0.21 c	1.61 ± 0.01 c	2.83 ± 0.01 c	1.05 ± 0.01 a
B10P2	99.38 ± 1.52 d	13.955 ± 1.2 b	14.78 ± 0.11 d	1.81 ± 0.03 e	3.25 ± 0.03 e	1.30 ± 0.02 d
B15P2	118.53 ± 0.77 f	16.25 ± 0.87 c	15.51 ± 0.33 e	1.74 ± 0.02 d	3.40 ± 0.04 f	1.37 ± 0.03 e

* Different letters in the first part of the table “a–c” in the second part “a–f”. significant difference in average values (*p* ≤ 0.05).

**Table 5:** Quantity and quality profiles of polyphenols in bread with a share of the preparations.

Sample	Content (mg/100 g d.m)										
	Hydroxybenzoic acid										
	GA	VA		PA		SYA	DA		PAL		EA
Control	0.28 ± 0.01 a *	nd		nd		0.35 ± 0.01 d	1.19 ± 0.01 d		6.30 ± 0.03 e		nd
B5P1	2.10 ± 0.04 c	nd		nd		0.28 ± 0.01 c	1.03 ± 0.01 c		6.00 ± 0.02 cd		0.28 ± 0.01 a
B10P1	2.50 ± 0.01 d	nd		nd		0.21 ± 0.02 b	1.15 ± 0.04 d		5.55 ± 0.02 a		0.48 ± 0.01 c
B15P1	2.93 ± 0.07 e	nd		0.46 ± 0.01 b		0.09 ± 0.01 a	1.07 ± 0.02 c		5.99 ± 0.03 c		0.36 ± 0.03 b
B5P2	1.25 ± 0.08 b	nd		nd		nd	1.00 ± 0.03 c		5.99 ± 0.02 c		0.54 ± 0.04 d
B10P2	1.35 ± 0.03 b	nd		nd		nd	0.75 ± 0.02 a		6.06 ± 0.02 d		0.32 ± 0.02 b
B15P2	2.57 ± 0.06 d	nd		0.34 ± 0.01 a		0.22 ± 0.03 b	0.88 ± 0.01 b		5.75 ± 0.03 b		0.40 ± 0.04 cd
	**Hydroxycinnaminic acid**										
	**CA**		**FA**		**PCA**			**CLA**		**SIA**	
Control	nd		1.35 ± 0.1 c		nd			nd		0.70 ± 0.01 c	
B5P1	nd		1.18 ± 0.08 b		nd			nd		0.56 ± 0.04 a	
B10P1	nd		1.05 ± 0.04 a		0.07 ± 0.01 a			nd		0.55 ± 0.02 a	
B15P1	0.25 ± 0.02 b		1.05 ± 0.08 a		0.15 ± 0.02 c			nd		0.64 ± 0.02 b	
B5P2	nd		1.30 ± 0.03 c		nd			nd		0.55 ± 0.04 a	
B10P2	nd		1.19 ± 0.03 b		nd			nd		0.52 ± 0.04 a	
B15P2	0.15 ± 0.01 a		1.02 ± 0.06 a		0.10 ± 0.01 b			nd		0.52± 0.05 a	
	**Flavonols and flavanols**										
	**Q3Gal**		**Q3Glu**		**Q3R**			**Q**		**E**	
Control	nd		nd		nd			nd		nd	
B5P1	0.03 ± 0.01 a		nd		0.08 ± 0.01 a			nd		nd	
B10P1	0.03 ± 0.01 a		nd		0.15 ± 0.02 b			0.15 ± 0.02 a		nd	
B15P1	0.04 ± 0.01 a		nd		0.15 ± 0.01 b			0.26 ± 0.03 c		nd	
B5P2	nd		nd		0.06 ± 0.02 a			0.16 ± 0.01 a		nd	
B10P2	nd		nd		0.04 ± 0.01 a			0.20 ± 0.01 b		nd	
B15P2	0.04 ± 0.01 a		nd		0.16 ± 0.01 b			0.17 ± 0.01 a		nd	

GA—gallic acid, VA—vanillic acid, PA—protocatechuic acid, SYA—syringic acid, DA—2,5-dihydroxybenzoic acid, PAL—protocatechuic aldehyde, EA—ellagic acid, CA—caffeic acid, FA—ferulic acid, PCA—p-coumaric acid, CLA—chlorogenic acid, SIA—sinapic acid, Q3Gal—quercetin 3-O-galactoside, Q3Glu—quercetin 3-O-glucoside, Q3R—quercetin 3-O-rutinoside (rutin), Q—quercetin, E—epicatechin. nd—Not detected. * Different letters in a–c significant difference in average values (*p* ≤ 0.05).

**Table 6:** Content of B vitamins in bread with a share of the preparations (mg/100 g d.m.).

Sample	B1	B2	B3	B6	Total
Control	0.07 ± 0.01 a *	1.39 ± 0.01 a	0.70 ± 0.01 b	nd	2.16 ± 0.03 a
B5P1	0.13 ± 0.01 b	1.62 ± 0.01 d	0.45 ± 0.01 a	0.11 ± 0.01 b	2.31 ± 0.04 b
B10P1	0.14 ± 0.01 b	1.88 ± 0.03 e	0.80 ± 0.05 c	0.13 ± 0.03 b	2.95 ± 0.11 e
B15P1	0.15 ± 0.03 b	2.21 ± 0.01 f	0.98 ± 0.01 d	0.14 ± 0.01 b	3.48 ± 0.06 f
B5P2	0.13 ± 0.01 b	1.90 ± 0.01 e	0.46 ± 0.01 a	0.11 ± 0.02 b	2.60 ± 0.05 c
B10P2	0.14 ± 0.01 b	1.57 ± 0.01 c	0.82 ± 0.02 c	0.12 ± 0.03 b	2.65 ± 0.07 c
B15P2	0.16 ± 0.02 b	1.46 ± 0.01 b	0.99 ± 0.01 d	0.13 ± 0.01 b	2.74 ± 0.05 d

nd—not detected. * Different letters in a–f significant difference in average values (*p* ≤ 0.05).

**Table 7:** Characteristics of bread with the studied preparations.

Sample	Specific Volume [g/cm^3^]	Bread Yield [%]	Crumb Moisture [%]		Hardness [N]		Cohesiveness	
			Day of Baking	48 h after Baking	Day of Baking	48 h after Baking	Day of Baking	48 h after Baking
Control	2.37 ± 0.06 c	122.0 ± 0.8 a	44.2 ± 0.1 ab	44.1 ± 0.7 a	10.0 ± 0.0 a	16.0 ± 0.3 a	0.732 ± 0.004 a	0.607 ± 0.024 a
B5P1	2.34 ± 0.19 c	123.1 ± 0.8 b	44.6 ± 0.6 b	44.6 ± 0.3 a	10.5 ± 0.6 a	15.4 ± 0.3 a	0.749 ± 0.011 abc	0.566 ± 0.037 a
B10P1	2.11 ± 0.10 b	123.3 ± 0.3 b	43.5 ± 0.4 a	44.2 ± 0.7 a	14.3 ± 0.3 cd	23.4 ±1.1 b	0.749 ± 0.008 abc	0.563 ± 0.043 a
B15P1	1.98 ± 0.08 a	123.2 ± 0.3 b	43.7 ± 0.8 a	44.4 ± 0.2 a	15.5 ± 1.4 d	26.1 ± 0.0 c	0.742 ± 0.003 ab	0.579 ± 0.033 a
B5P2	2.22 ± 0.04 b	123.5 ± 0.8 b	44.6 ± 0.5 b	44.7 ± 0.5 a	10.2 ± 0.5 a	17.2 ± 1.8 a	0.767 ± 0.004 c	0.554 ± 0.030 a
B10P2	2.13 ± 0.15 b	123.6 ± 0.8 b	43.9 ± 0.4 ab	43.9 ± 0.7 a	11.5 ± 0.5 ab	22.8 ± 1.0 b	0.761 ± 0.012 bc	0.557 ± 0.040 a
B15P2	1.97 ± 0.17 a	124.5 ± 0.0.6 c	44.1 ± 0.5 ab	43.9 ± 0.4 a	13.2 ± 1.2 bc	23.2 ± 0.9 b	0.765 ± 0.016 bc	0.561 ± 0.027 a

Different letters in a–d significant difference in average values (*p* ≤ 0.05).

**Table 8:** Crumb color of the tested bread samples.

Sample	L* (D65)	a* (D65)	b* (D65)	ΔE
Control (Standard)	52.42 ± 1.16 c	8.94 ± 0.17 c	22.13 ± 0.21 c	-
B5P1	49.39 ± 1.20 ab	8.84 ± 0.13 bc	21.56 ± 0.31 b	3.10 ± 1.17 bc
B10P1	48.19 ± 0.63 a	8.71 ± 0.16 b	21.39 ± 0.25 b	4.30 ± 0.66 d
B15P1	48.44 ± 1.03 a	8.63 ± 0.22 b	21.51 ± 0.09 b	4.04 ± 1.01 cd
B5P2	51.53 ± 1.47 c	8.41 ± 0.09 a	21.58 ± 0.32 b	1.76 ± 0.32 a
B10P2	50.10 ± 0.45 b	8.30 ± 0.11 a	21.07 ± 0.15 a	2.63 ± 0.43 ab
B15P2	49.45 ± 0.51 ab	8.34 ± 0.11 a	21.36 ± 0.26 ab	3.13 ± 0.52 bc

Different letters in a–d significant difference in average values (*p* ≤ 0.05).

**Table 9:** Volatile compound content (%) of the extruded formulations.

Compound	Compound Class	P1	P2
Ethanol	Alcohols	10.19 ± 0.09 a	12.48 ± 0.06 b
2-Methyl-1-propanol		7.59 ± 0.01 b	4.33 ± 0.01 a
1-Hexanol		7.14 ± 0.01 b	6.04 ± 0.10 a
2-Phenylethanol		3.73 ± 0.05 a	3.72 ± 0.01 a
1-Nonanol		3.69 ± 0.01 a	4.35 ± 0.01 b
2-Methylpropanal	Aldehydes	nd	0.77 ± 0.01 a
3-Methylbutanal		nd	0.90 ± 0.01 a
Hexanal		0.72 ± 0.01 a	1.17 ± 0.01 b
Furfural		nd	nd
2-Decenal		5.38 ± 0.01 b	4.43 ± 0.01 a
2.4-(E.E)-Decadienal		3.79 ± 0.01 a	4.65 ± 0.01 b
Dodecanal		3.80 ± 0.02 b	1.51 ± 0.01 a
2-Methylpropanoic acid	Carboxylic acids	1.75 ± 0.01 a	1.95 ± 0.01 b
3-Methylbutanoic acid		6.61 ± 0.01 b	5.20 ± 0.02 a
Pentanoic acid		3.80 ± 0.01 b	3.47 ± 0.01 a
p-Methoxybenzoic acid		nd	0.59 ± 0.01 a
Dodecanoic acid		1.81 ± 0.01 b	1.66 ± 0.01 a
Tridecanoic acid		8.04 ± 0.01 b	7.34 ± 0.02 a
Pentadecanoic acid		1.41 ± 0.01 a	1.91 ± 0.01 b
Ethyl acetate	Esters	1.51 ± 0.01 a	1.63 ± 0.01 b
Isopropyl acetate		nd	0.65 ± 0.01 a
Phenylethyl acetate		1.22 ± 0.01 a	1.35 ± 0.01 ab
Ethyl dodecanoate		6.52 ± 0.07 a	6.63 ± 0.07 b
Benzyl phenyl acetate		1.03 ± 0.01 b	0.60 ± 0.01 a
Benzyl benzoate		3.92 ± 0.01 a	5.27 ± 0.02 b
2-Phenylethyl phenylacetate		0.72 ± 0.01 a	nd
Butane-2.3-dione	Ketones	3.45 ± 0.01 b	3.21 ± 0.01 a
Pentan-2-one		1.38 ± 0.01 b	1.21 ± 0.01 a
5-Propyldihydro-2(3H)-furanone	Lactones	2.69 ± 0.03 a	2.75 ± 0.01 b
delta-Decalactone		0.81 ± 0.01 a	1.87 ± 0.01 b
4-Undecanolide		nd	0.63 ± 0.01 a
Guaiol	Phenols	1.10 ± 0.01 a	nd
Methylpyrazine	Pyrazines	3.71 ± 0.04 b	1.97 ± 0.01 a
2.5-Dimethylpyrazine		1.38 ± 0.03 a	3.94 ± 0.01 b
2.6-Dimethylpyrazine		1.12 ± 0.02 a	1.82 ± 0.01 b

nd—not detected; different letters in a,b significant difference in average values (*p* ≤ 0.05).

## Data Availability

The original contributions presented in the study are included in the article, further inquiries can be directed to the corresponding author.
